# Dental Education

**Published:** 1876-11

**Authors:** B. F. Coy


### DENTAL EDUCATION.

BY DR. B. F. COY,

*Read before the Maryland State Dental Society*

To me has been assigned the task of preparing a paper on dental education. I will not shrink from this duty, for several reasons: In the first place it is a most important subject, and one which most certainly will decide the future of our profession. "As the twig is bent, the tree is inclined." Another reason is, that considering the earnest men of this or any other profession as like a hive of bees, upon each individual of which depends the welfare of the association, no individual has the right to shirk the particular duty assigned to him. I have for the last several years given especial attention to the subject of dental education, and having in one paper, read before the American Dental Convention, which met at Saratoga last year, discussed the question, I almost feel myself a bankrupt, having in this paper exhausted my whole supply of ammunition. Again, however, at the last meeting of the American Dental Convention, which met in August of this year at Long Branch, I had to call the attention of that meeting to some new theories and ideas which were separating the profession on this subject. But, as the presiding officer of that convention, I did not feel at liberty to intrude my opinions or to enter into the debates. I simply presented the different views to be discussed, without leaning to the one or the other, or endeavoring to prejudice any. I feel, however, in this society that I not only have a right to ventilate these matters, but that it becomes a positive duty. In this association I have naturally more individual interest, and must, therefore, take a firmer and more decided stand. Here I am relieved of all sense of delicacy, which as presiding officer I felt at Long Branch, and here I am a high private in the rear ranks battling for what is best and most essential to our welfare and preservation. I shall take the liberty of quoting from my paper read at Long Branch these differing views, and I then propose to take them up seriatim and then discuss them. In reviewing the efforts of the profession generally in the past year to keep up with the progressive spirit of the age, I said:

"Let us look for a moment at what has *been done.* As regards the question of dental education and the elevation of the standard of proficiency or graduation, there has been apparently almost an entire upheaving, and we are consequently in a condition of revolution and chaos.

"We have among us honest men and true, who are advocating with might and main that our dental degrees shall be dropped altogether, and that we shall only be known as graduates of medical schools, the specialty of dentistry being classed, like other medical specialties, all under the one absorbing and controlling title of M. D. We have other men equally honest and true who are fighting most earnestly against this movement and believe that it would undo all that has been so far accomplished.

"We have, too, our dental schools differing from each other in regard to the best regime of dental education and graduation. Some are in favor of extending the time of preparation, and think it best after the year seventy-seven to take no amount of knowledge or previous preparation as the equivalent of a college course. Some are in favor of a three years' course; others two, and others again which hold to the opinion that a diploma should be conferred whenever merited, regardless of every other consideration; and yet these different schools are all honest and true and are all working for the best. But each one believes its own regime the proper course to pursue. Again: Some of our dental schools would be very unwilling to have a diploma conferred by any other power than a regularly organized faculty. Who, say they, are so competent to judge of a candidates proficiency as his teachers? Others, again, have thought it most advisable to have a board of directors from the best men in the dental profession, and to this board should be delegated the whole power of conferring degrees. Others, again, think that probably the most important step that can be taken towards the elevation of the standard of dental education is to demand a preliminary examination before matriculation, and thus in the very onset begin to get rid of the chaff.

"Now all these different opinions come from men who are honest and true, but, as you perceive, we are in a state of chaos, which possibly time alone will elaborate into certainty and give us some fixed system; but before these questions can be rightfully disposed of for the good of all concerned, we must earnestly counsel together, and each man must unselfishly do all in his power to promote the general welfare, and out of all this chaos there will some day or other come such a creation as will satisfy us all.

"We can not all think alike now, but let us refrain from dogmatism, and recollect that nothing human is immaculate. Don't be too certain in your own opinions, and learn never to under rate the capacity or integrity of those who disagree with you.

"I have presented these matters to the convention because I believe them to be vital questions, involving to a great extent the best interests of the profession, and I sincerely hope and recommend that they will be freely and faithfully discussed.

"Let each man in the convention, with malice apart, express himself freely and give us the benefit of his own honest convictions. Let us have a "fair field and no favor." It is in this way only that we can become enlightened. We need, sorely need, this counseling together. The profession is getting too much scattered on these questions, and like lost sheep need bringing together. 'In unity there is strength, I hope these matters will be fully considered."

In regard to the first question proposed—whether our dental degrees shall be yielded up and sacrificed on the alter of that portion of general medicine which is covered or represented by the symbol M. D.,—I might say much, but as I have learned that this matter will be brought forward in a paper to be read by another brother at this meeting, to him I shall yield the floor, feeling confident that it will be handled in a most able manner.

The second proposition is simply "proficiency versus time." Whether a certain amount of knowledge and skill shall alone constitute the requirements which shall justify the conferment of a degree, or whether a candidate for graduation must have attended a certain number of college courses, and have been engaged in the pursuit of his profession a certain length of time. Really, in my own judgment, there is but one side to this question. If all men were born alike, with the same amount of mechanical taste, the same readiness of observation and clear perception, the same amount of application, and the same ease and facility in acquiring nice manipulative touch, then we might at once conclude that all that was or should be required would be the time test. When all things being equal in the commencement, and alike influenced and improved according to the number of hours which had elapsed since the commencement, then, of necessity, such a length of time would invariably bring the required amount of proficiency up to the standard agreed upon, and dental education would be reduced to a simple mathematical proposition.

But do we find this equality? All will answer at once, no. Then how is it possible thus to equalize the attainments of individuals?

Does a dental diploma mean to certify to nothing more than the fact that its recipient has been engaged in the pursuit of it so many years, months, days, and hours? Is this what a diploma means? We think not. It does or should mean that its recipient has acquired a sufficient amount of knowledge and skill to *deserve* this certificate of excellence. Is it possible that any man in the world would say that Dr. Hunt, one of the brightest ornaments in our profession, (he will pardon me for using his name,) who is without a dental degree, ought to be put, should he desire a diploma, on the back bench of a college lecture room, and in company with mere children, who have come to school with clean slates yet to learn their first lesson? Would not this be a first-class farce? Then why should not Dr. Hunt be placed on the rear seats? Simply because his attainments are such that he deserves more consideration, he is by these very attainments ripe for his degree, should he desire it, and there is no college in the country that would not be proud to reckon him among her alumni. Well, if this principle will not apply to Dr. Hunt, which I admit is an extreme case, is it a correct principle? Should not a diploma be conferred whenever merited? Then why mention time as a requisite? Why not put the standard where it should be, and require every candidate to measure up to it? When he has done so, he is entitled to his diploma and it should be given to him. What then has one year or ten years to do with the question? One college course or fifty college courses? The question certainly is not how long the candidate has been in pursuit of his degree; but what is the amount of knowledge acquired. I shall not discuss the abuses to which this system might give rise.

There is nothing so good that abuses may not follow. But this does not alter the principle of right and justice in the matter. Because some college might take advantage of this system, and, like a late medical school of Philadelphia, avalanche this whole country and Europe, too, with its bought and sold diplomas, yet it would not affect the principle or system one iota. I could say much more on this subject, but, in the first place, I do not think it demands further discussion, and, secondly, I have something to say on other matters, and do not wish to make this paper to long. The next question I shall discuss is, Who should confer dental degrees? Whether a faculty, or a well appointed board of directors or regents, carefully selected from the dental profession—men eminently qualified to judge of the fitness of a candidate for the degrees, and, at the same time, above any possible suspicion of being governed by favoritism, or actuated by self-interest,—men not having one single cent at stake in the financial or pecuniary condition of the college. I know much may be said on the other side, and I do not mean to impugn the honesty or integrity of any dental faculty in the country. But I *do say*, that when the whole power of granting or not granting diplomas is vested in outside reliable members of the profession, whose only real interest is to prevent improper graduations, and when they alone make the final examinations, that the degree of a college organized under this system must of necessity be honorable and honored.

I appeal to all of you gentlemen who are present. What would be your object in accepting a regency in a school so organized? It could not be self-interest, because you would not only receive no pay, but you would have to lose your valuable time, and pay your own expenses. You could then have but one object in accepting such a position, and that would be to see and know for yourselves that no degree was improperly conferred. Very much more might be said on this subject, but I must not take up your time, and shall consequently pass to the next question.

Shall our colleges accept any and all sorts of material that may come to them in the shape of students who can bring with them only the amount of the college fees? We think not. If it is desirable to raise the standard of dental education and graduation, can it be done except through intelligence and attainment? Have any of the other sciences or professions been progressive through ignorance? It is true, unfortunately, that you can take a first-class ignoramus and teach him dentistry as a trade. Is this what is desired or wanted in a profession? What would be the use of a so-called student (one even ignorant of his own language) attempting to enter the labyrinth of anatomy, physiology, chemistry, pathology, therapeutics, materia medica, and the principles of these collateral branches as applied to scientific dentistry? It is, or would be, another first-class farce. Can you build a stone edifice except of stone? Is not the system unfair to the teachers of dentistry, and is it not unjust to the marticulant? You have deliberately taken his money,—what does he get in return? Does he get his diploma? If so, I would not give much for such a diploma.

Dental schools under this system have aped medical schools.

How stands to-day the degree of M. D. in this country, compared with the degrees conferred under a different system —for instance, the degrees of Oxford, Cambridge, Yale, Harvard, Princeton, the University of Virginia, etc? Do they not "grow small and beautifully less?" We think that before matriculation there should be an examination as to previous preparation, in common English branches at least. This would be fair and just, both to instructor and matriculant. I know it may be said that great men have been made from very obscure commencements, and it might be argued that a preliminary examination would be a great wrong in depriving such a student of the privilege of a chance for improvement; but in doing so you would forget the fact that those self-made men are the exceptions, and you would also forget the great wrong perpetrated on the majority by such laxity. I will close by thanking you for your patient attention, and requesting that these questions be fairly and freely discussed in this meeting, that we may be led to the right if in error.—*Proceedings of Md. State Dental Society.*

